# Advances in spatial transcriptomics and its application in the musculoskeletal system

**DOI:** 10.1038/s41413-025-00429-w

**Published:** 2025-05-16

**Authors:** Haoyu Wang, Peng Cheng, Juan Wang, Hongzhi Lv, Jie Han, Zhiyong Hou, Ren Xu, Wei Chen

**Affiliations:** 1 Department of Orthopedic Surgery, Hebei Medical University Third Hospital, Shijiazhuang, Hebei China; 2 Key Laboratory of Biomechanics of Hebei Province, Shijiazhuang, Hebei China; 3 NHC Key Laboratory of Intelligent Orthopedic Equipment, Shijiazhuang, Hebei China; 4 Department of Orthopedics, Union Hospital, Tongji Medical College, Huazhong University of Science and Technology, Wuhan, Hubei China; 5 State Key Laboratory of Cellular Stress Biology, Cancer Research Center, School of Medicine, Faculty of Medicine and Life Sciences, Xiamen University, Xiamen, Fujian China; 6 The First Affiliated Hospital of Xiamen University-ICMRS Collaborating Center for Skeletal Stem Cells, State Key Laboratory of Cellular Stress Biology, School of Medicine, Faculty of Medicine and Life Sciences, Xiamen University, Xiamen, Fujian China

**Keywords:** Bone, Pathogenesis

## Abstract

While bulk RNA sequencing and single-cell RNA sequencing have shed light on cellular heterogeneity and potential molecular mechanisms in the musculoskeletal system in both physiological and various pathological states, the spatial localization of cells and molecules and intercellular interactions within the tissue context require further elucidation. Spatial transcriptomics has revolutionized biological research by simultaneously capturing gene expression profiles and in situ spatial information of tissues, gradually finding applications in musculoskeletal research. This review provides a summary of recent advances in spatial transcriptomics and its application to the musculoskeletal system. The classification and characteristics of data acquisition techniques in spatial transcriptomics are briefly outlined, with an emphasis on widely-adopted representative technologies and the latest technological breakthroughs, accompanied by a concise workflow for incorporating spatial transcriptomics into musculoskeletal system research. The role of spatial transcriptomics in revealing physiological mechanisms of the musculoskeletal system, particularly during developmental processes, is thoroughly summarized. Furthermore, recent discoveries and achievements of this emerging omics tool in addressing inflammatory, traumatic, degenerative, and tumorous diseases of the musculoskeletal system are compiled. Finally, challenges and potential future directions for spatial transcriptomics, both as a field and in its applications in the musculoskeletal system, are discussed.

## Introduction

Since the advent of next-generation sequencing in 2005 (ref. ^1^), transcriptomics research has made substantial advances. Bulk RNA sequencing (RNA-seq) provides information on gene expression, RNA structure, and protein translation, and is capable of identifying new genes and deciphering signal networks related to physiological states and pathological processes.^2,3^ However, RNA-seq only detects the average gene expression levels of mixed cells, masking phenotypic details of individual cells and the differences in transcriptomes between cells, leading to the potential dilution and oversight of important transcripts in specific cell types.^4^ To overcome this limitation, single-cell RNA sequencing (scRNA-seq) was introduced by Tang et al.^5^ in 2009, achieving the quantification of transcriptomes within individual cells for the first time. It provides a powerful tool for studying cellular heterogeneity, characterizing new cell types and states, and elucidating regulatory networks between cell clusters.^6^ However, scRNA-seq requires the harvest of single live cells from tissues without inducing cell stress or death. During cell separation, the disruption of intercellular connections and changes in the external microenvironment can lead to alterations in the internal transcriptome.^4^ Additionally, the hardness of bone and cartilage tissues and the specific cellular morphology of muscle cells present challenges in preparing single-cell suspensions from these tissues.^7^

Despite the unique data provided by RNA-seq and scRNA-seq for exploring tissue cellular heterogeneity, transcriptomic analysis by either of these techniques results in loss of the spatial context of cells,^8^ which is closely related to biological function.^9^ For example, from the enthesis with higher calcification to the tendon mid-body, and then to the myotendinous junction, the spatial organization of cells within the tendon is crucial for their ability to bear and transmit tensile forces.^10^ Similarly, the transcriptional heterogeneity of subpopulations of skeletal stem and progenitor cells (SSPCs) may depend on their diverse spatial positioning and ecological niches within the bone marrow. Even subtle changes in localization or intercellular crosstalk of SSPCs in the bone marrow microenvironment can profoundly impact their functional state or cell fate.^11^ Furthermore, although researchers can theoretically infer potential mechanisms based on receptor–ligand interactions from RNA-seq and scRNA-seq data, the biological feasibility of such mechanisms still requires further validation, specifically whether the interacting cells are in close spatial proximity and express the necessary genes in that spatial context.^12^ All these points emphasize the significant role of spatial location information in biomedical research.

The concept of spatial transcriptomics (ST) was first introduced in 2016 by the Lundeberg research group.^13^ Unlike traditional sequencing methods, it allows for the quantification and localization of the transcriptome while preserving the original spatial context. This approach provides data on associated cellular gene expression levels and spatial location information, creating a visual spatial transcriptome map that integrates with tissue morphological characteristics. For example, ST has been used to construct the first spatiotemporal transcriptomic atlas of human embryonic limb development.^14^ Moreover, ST helps to decipher the true gene expression of cells in situ within tissues, avoiding biases in detection caused by the loss of certain cell types or changes in cell transcriptomes during the single-cell dissociation process. For example, despite the important role adipocytes play in the bone marrow microenvironment, their fragility and large size make them difficult to analyze by scRNA-seq.^15^ Similarly, the multinucleated nature of mature osteoclasts^16^ and muscle fibers,^17^ along with the fragile nature and large cell volume of true hypertrophic chondrocytes,^18^ pose significant challenges for single-cell isolation and analysis. The spatial location information provided is advantageous for elucidating physiological and pathological mechanisms. At the subcellular level, the spatial localization of messenger RNA (mRNA) plays a crucial role in precisely controlling protein synthesis and function, helping to elucidate the spatial regulation of gene activity. At the cellular level, clarifying the spatial positions of cells within tissues aids in identifying cell types, defining cell functions, and deciphering the spatial organization of cells and their intercellular interaction networks.^19,20^ For example, ST has been utilized to analyze the cellular composition, spatial organizational structure, and intercellular crosstalk within the microenvironment of the SSPC niche in the bone marrow.^12^ As ST introduces a spatial dimension to gene expression studies, it offers a novel perspective and methodology for biomedical research, earning it the title of “Method of the Year 2020” by Nature Methods.^21^

ST technologies can be divided into two main categories based on RNA detection strategies: imaging-based ST technologies and sequencing-based ST technologies.^9^ With the continuous development of microscopic imaging and processing strategies and sequencing technologies, improvements in sequencing cost-effectiveness, and ongoing enhancements in computational strategies, the capabilities for data acquisition and analysis in ST have rapidly advanced. Currently, ST technologies have achieved unbiased whole-transcriptome analysis,^13^ nanoscale spatial resolution,^22^ and 3D construction of transcriptomic landscapes,^23,24^ among other multidimensional functionalities. In terms of applications, ST has been widely used in various fields including embryonic development,^25–29^ oncology,^30,31^ immunology,^32,33^ and neuroscience.^34,35^ However, the application of ST to the musculoskeletal system is still in the developmental stage. While Feng et al. provided a valuable overview of single-cell and spatial omics in musculoskeletal disorder research,^36^ a comprehensive review specifically focused on the advances and challenges of ST in this field remains lacking.

This article provides a brief overview of the development of ST technologies, focusing on the latest technological advances and widely-utilized representative technologies, along with a brief workflow for integrating ST into musculoskeletal system research. We summarize research progress in revealing the physiological mechanisms of the musculoskeletal system, particularly during developmental processes, as well as new findings and achievements in the study of inflammatory, traumatic, degenerative, and tumorous diseases of the musculoskeletal system. Finally, we discuss the current challenges and future developments, including challenges and prospects for the application of ST to the musculoskeletal system, the 3D landscape of the transcriptome, spatial multi-omics and spatiotemporal omics, and the application of artificial intelligence (AI) in ST.

## Developments and classification of ST

Previous reviews have already provided a high-quality summary of ST data acquisition technologies.^9,20,37–41^ Therefore, this section provides only a brief overview of these technologies, focusing on the latest technological innovations and widely-adopted representative methods.

### Imaging-based ST technologies

Imaging-based ST technologies (Table 1) include in situ hybridization (ISH) techniques, which utilize labeled probes containing complementary sequences to detect target RNA, and in situ sequencing (ISS) techniques that directly sequence RNA in its native tissue context.

**Table 1:** Imaging-Based ST Technologies

Method	Inventor	Year established	Sample type	Advantages	Limitations	Gene detection efficiency	Targeted/ transcriptome-wide	Spatial resolution	Commercial platform (vendor)	References
*ISH-based*										
smFISH	Femino et al.	1998	FF/FFPE	High sensitivity	Low throughput	Nearly 100%	Targeted	Subcellular	RNAscope (Bio-Techne)	^44,45^
seqFISH	Lubeck et al.	2014	FF/FFPE	Multiplexing	High cost, high error, optical crowding, limited view	84%	Targeted	Subcellular	Molecular Cartography (Resolve Biosciences)	^47,225^
MERFISH	Chen et al.	2015	FF/FFPE	Multiplexing, improved detection robustness	High cost, limited view	80%–95%	Targeted	Subcellular	MERSCOPE (Vizgen)	^49,226^
seqFISH+	Eng et al.	2019	FF	Higher level of multiplexing, each image displays part of the transcripts, reducing optical crowding	High cost, time-consuming, limited view: analysis of small tissue profiles, only 1 mm^2^	49%	Targeted	Subcellular	NA	^48,50^
EEL FISH	Borm et al.	2022	FF	Multiplexing, high throughput, low cost, gapless RNA capture, eliminates tissue background impact	Low detection sensitivity	2.6%–13.2%	Targeted	Subcellular	Rebus Esper (Rebus Biosystems)	^50^
CosMx SMI	Nanostring	2022	FF/FFPE	Automated equipment, targeted detection of RNA and proteins at the spatial level, high signal-to-noise ratio	Low throughput	One or two copies per cell	Targeted	Subcellular	CosMx SMI (Nanostring)	^51^
*ISS-based*										
FISSEQ	Lee et al.	2014	FF/FFPE	Whole transcriptome analysis	Limited read length, extremely low detection efficiency, high proportion of rRNA reads, low throughput, expensive, optical crowding	< 0.005%	Transcriptome-wide	Subcellular	RC2 (ReadCoor)	^56,227^
STARmap	Wang et al.	2018	FF	High signal-to-noise ratio, high sensitivity, high precision, capable of resolving thick tissue sections	Low throughput, limited view	Slightly better than scRNA-seq	Targeted	Subcellular	Plexa In Situ Analyzer (Stellaromics)	^54^
Exseq	Alon et al.	2021	FF/FFPE	Optional targeted and non-targeted, nanoscale subcellular spatial resolution	Long imaging time	~43.4% (Targeted)	Targeted or transcriptome-wide	Subcellular	NA	^22^
Xenium	10x Genomics	2023	FF/FFPE	High detection sensitivity for low-level expressed genes, high specificity, fast data output capability	Platform capable of combined proteomic analysis pending release	1.4 times higher than scFFPE-seq	Targeted	Subcellular	Xenium (10x Genomics)	^53^
Electro-seq	Li et al.	2023	Live cells	Paired detection of 3D transcriptional states and electrophysiological states	The causal relationship between gene expression and electrophysiology still needs further exploration	NA	Targeted	Cellular	NA	^55^

#### ISH-based ST technologies

ISH techniques have evolved from early radioactive methods^42,43^ to fluorescence-labeled approaches, which have been continuously refined over the past decades.^44,45^ RNAscope, as one of the earliest commercially-available ST platforms, detects a limited number of genes with high sensitivity at subcellular resolution.^46^ To enhance RNA detection throughput, researchers have employed multiplexing encoding strategies, using unique fluorescent sequences or binary codes corresponding to individual RNAs. SeqFISH,^47^ seqFISH+,^48^ MERFISH,^49^ EEL FISH^50^ and NanoString’s Spatial Molecular Imaging (SMI) technology^51^ have significantly expanded the scale of target detection. MERFISH employs a unique binary encoding strategy integrated with error correction schemes, significantly enhancing the robustness of transcript recognition.^49^ EEL FISH uses electrophoresis to move RNA to a capture plane with minimal lateral dispersion, reducing tissue background interference while decreasing the time required for thick tissue z-axis imaging, albeit at the cost of losing axial resolution.^50^ SMI utilizes detection panels covering up to 18 000 genes and is the first imaging-based ST technology claiming near-whole transcriptome coverage in human or mouse samples. Additionally, it enables targeted multi-omics detection of RNA and proteins.^51^ Overall, the notable advantage of ISH-based ST technologies lies in their ability to detect low-abundance transcripts with high sensitivity while directly capturing their precise spatial information.

#### ISS-based ST technologies

Targeted ISS techniques utilize padlock probes and rolling-circle amplification to achieve in situ sequencing of target RNA.^52^ Xenium can detect low-abundance genes with high sensitivity and specificity and has rapid data output capabilities.^53^ The newly-introduced Xenium Prime 5 K assays can simultaneously detect up to 5 000 genes in human or mouse samples. Additionally, researchers can design fully customizable gene panels containing up to 480 genes to meet specific research needs. The commercialized version of STARmap, Plexa In Situ Analyzer, enables the mapping of spatial gene expression patterns in thick tissue samples with intact structural integrity, providing high-resolution 3D multi-omics perspectives.^54^ Electro-seq, a yet-to-be-commercialized technology, integrates chronic electrophysiological recordings with the construction of 3D transcriptome landscapes, providing a promising tool for characterizing cell states and developmental trajectories in electrogenic tissues, such as skeletal muscles.^55^

The introduction of non-targeted ISS techniques has facilitated a shift from targeting only known sequences to exploring unknown genes. FISSEQ^56^ and ExSeq^22^ are capable of unbiased whole-transcriptome analysis, albeit at the cost of low gene detection efficiency. Compared to ISH techniques, ISS-based ST technologies enable the detection of both targeted and non-targeted transcripts, offer a higher signal-to-noise ratio, and possess the capability to detect single-nucleotide variations.^57^ However, due to inefficient reverse transcription steps and the low ligation efficiency of padlock probes, ISS-based ST technologies exhibit lower detection sensitivity, particularly when employing multiplexing strategies.^57,58^

#### Advantages and limitations of imaging-based ST technologies

Imaging-based ST technologies share several common advantages. First, high sensitivity and subcellular resolution are two key strengths. However, gains in sensitivity and spatial resolution come at the cost of detecting fewer transcripts.^59^ Second, these technologies are broadly applicable to formalin-fixed and paraffin-embedded (FFPE) samples, which is particularly valuable in musculoskeletal research, especially when working with hard tissues such as bone. This will be elaborated upon in detail later in the text. Like their advantages, the limitations of imaging-based ST technologies are also pronounced. First, the targeted strategy restricts new discoveries of genes and their spatial positions that are not included in the prespecified target panel. Second, imaging-based ST technologies often entail high temporal and financial costs, requiring expensive and complex microscopes or staining apparatus. Third, the maximum imaging area of currently available technologies, provided by Xenium at 4.72 cm², limits their applicability to long tissue sections, such as the longitudinal sections of tendons. Fourth, the imaging data from a single sample can reach several hundred gigabytes, posing significant challenges for data storage, processing, and sharing.

### Sequencing-based ST technologies

Sequencing-based ST technologies (Table 2) record spatial positions by selecting regions of interest (ROIs) or using spatial barcodes. Spatial barcodes can be organized in an array on the capture surface, printed onto tissues via microfluidic channels, or directly tagged to individual cells or nuclei.

**Table 2:** Sequencing-Based ST Technologies

Method	Inventor	Year established	Sample type	Advantages	Limitations	Gene detection efficiency	Targeted/ transcriptome-wide	Spatial resolution	Commercial platform (vendor)	References
*ROI-selection-based*										
LCM	Emmert-Buck et al.	1996	FF/FFPE	Highly suitable for FFPE, whole transcriptome analysis, high spatial resolution	Time-consuming, low throughput	NA	Targeted or transcriptome-wide	Cellular	LCM(Arcturus, PALM, Leica)	^60^
Geo-seq	Chen et al.	2017	FF	Robust, more sensitive than LCM	Low throughput, difficult to achieve single-cell spatial resolution	NA	transcriptome-wide	Multicellular	NA	^24^
GeoMx DSP	NanoString	2019	FF/FFPE	Multiplexing, suitable for FFPE samples, capable of combining multi-omics detection	Limited sensitivity	NA	transcriptome-wide^a^	Variable, minimum up to 10 μm	GeoMx DSP (NanoString)	^61^
*Spatial barcode-based*										
Visium	10× Genomics	2019	FF/FFPE	Unbiased whole transcriptome analysis, adaptable to FFPE tissues	Limited RNA capture efficiency, limited spatial resolution	>6.9%	transcriptome-wide^a^	55 μm	Visium (10× Genomics)	^13^
Slide-Seq	Rodriques et al.	2019	FF	Unbiased whole transcriptome analysis, high spatial resolution	Not suitable for highly heterogeneous samples, low capture efficiency, in situ sequencing is time-consuming	0.3%	transcriptome-wide	10 μm	NA	^66^
DBiT-seq	Liu et al.	2020	FF/FFPE	Unbiased whole transcriptome analysis, variable spatial resolution, suitable for FFPE tissues, detection of RNA and proteins in a spatial context	Limited spatial resolution	~15.5%	transcriptome-wide	Variable (10 μm, 25 μm, 50 μm)	NA	^68^
Slide-seqV2	Stickels et al.	2021	FF	Unbiased whole transcriptome analysis, high spatial resolution, improved capture efficiency	Capture efficiency remains low	Approximately 10 times higher than Slide-seq	transcriptome-wide	10 μm	Curio Seeker (Curio Biosciences)	^67^
Stereo-seq	BGI Genomics	2021	FF/FFPE	Unbiased whole transcriptome analysis, very high spatial resolution, large detection area	Capture efficiency remains low	Comparable to Visium	transcriptome-wide	220 nm	STOmics Stereo-seq, Stereo-seq OMNI (BGI Genomics)	^28,38,228^
XYZeq	Lee et al.	2021	FF	Unbiased whole transcriptome analysis, true single-cell resolution	Limited spatial resolution	NA	transcriptome-wide	500 μm (spot-to-spot center distance)	NA	^70^
sci-Space	Srivatsan et al.	2021	FF	Unbiased whole transcriptome analysis, true single-cell resolution	Limited spatial resolution	NA	transcriptome-wide	~222 μm (spot-to-spot center distance)	NA	^71^
xDBiT	Wirth et al.	2023	FF/FFPE	Unbiased whole transcriptome analysis, improved gene detection efficiency and throughput compared to DBiT-seq	Limited spatial resolution	Higher than DBiT-seq	transcriptome-wide	50 μm	NA	^69^
Visium HD	10×Genomics	2024	FFPE	Using about 18 000 probes for near-whole transcriptome level analysis, no gaps in capture area	Only compatible with human and mouse FFPE tissues	NA	transcriptome-wide^a^	2 μm	Visium HD (10× Genomics)	^229^

^a^Near-complete transcriptome coverage can only be achieved in human/mouse samples or evolutionarily similar species

#### ROI-selection-based ST technologies

Separation of ROIs through physical dissection or the application of optical or molecular markers represents a straightforward and effective strategy for obtaining spatial location information. Laser capture microdissection (LCM)^60^ is one of the representative techniques for physical dissection, utilizing ultra violet (UV) lasers to cut tissues or infrared radiation (IR) lasers to fuse tissues with a membrane to isolate ROIs. It is highly compatible with FFPE samples and extensively used in ST research. Geo-seq, which combines LCM with scRNA-seq, enables the exploration of cellular heterogeneity within ROIs.^24^ Using optical or molecular markers to designate ROIs avoids the impacts of physical dissection on the transcriptome. GeoMx Digital Spatial Profiler (GeoMx DSP)^61^ enables the quantitative analysis of RNA and proteins within ROIs in FFPE and FF samples by utilizing cleavable oligonucleotides and antibodies. In summary, the advantage of such technologies lies in the ability for researchers to select ROIs based on functional units of the tissue. However, the selection of ROIs is labor-intensive, requires prior knowledge, and carries the risk of potential selection bias. Moreover, these technologies typically do not offer single-cell spatial resolution.

#### Spatial barcode-based ST technologies

Since 2016, researchers have utilized spatial barcodes and unique molecular identifiers for the localization and quantification of RNA. *Spatial Transcriptomics (ST)*^13^ pioneered this approach, capturing transcripts at array sites containing spatial barcodes. Visium, an upgraded version of *ST* commercialized by 10x Genomics, has improved detection efficiency and spatial resolution. Visium is now compatible with FFPE samples^62^ and supports Nanopore long-read sequencing.^63^ Two improved versions of Visium, RNA-rescue ST (RRST)^64^ and spatial total RNA-sequencing (STRS),^65^ enable effective analysis of RNA from low-quality FF tissues and detection of a full spectrum of RNA, including non-coding RNA, respectively. The introduction of Slide-Seq^66^ enabled spatial resolution to reach near single-cell accuracy for the first time in such technologies. Slide-seqV2 (ref. ^67^) has undergone improvements in bead synthesis, array indexing, and library preparation, enhancing detection efficiency. Termed as the “ultra-wide-angle ten billion-pixel camera of life”, Stereo-seq^28^ uses DNA Nanoballs for unbiased whole-transcriptome detection at nanoscale resolution and high sensitivity in large fields of view. Notably, the cDNA generated by Stereo-seq requires sequencing on a specialized sequencer. Visium HD, recently released by 10x Genomics, achieves continuous tissue coverage without capture gaps, obtaining high-quality data at the micrometer scale.

Strategies that print spatial barcodes onto tissues via microfluidic channels effectively circumvent issues of RNA lateral diffusion during capture. DBiT-seq,^68^ as a representative technology of this strategy, enables spatial quantification of mRNA and proteins. xDBiT,^69^ an upgraded version of DBiT-seq, improves gene detection efficiency and throughput. Compared to capture array-based approaches, microfluidics-based ST technologies are more cost-effective.^58^

Due to the complexity of cell contours, a single capture area may capture transcripts from multiple cells, and transcripts within a single cell may contribute to multiple capture points. Therefore, performing actual single-cell level transcriptomic analysis is highly challenging. In XYZeq^70^ and sci-Space,^71^ spatial barcodes are used to mark individual cells or nuclei, rather than capture points, achieving true single-cell resolution at the expense of lower spatial resolution.

#### Advantages and limitations of sequencing-based ST technologies

Compared to image-based strategies, these techniques significantly improve throughput and whole transcriptome unbiased analysis capabilities. In addition, sequencing-based ST technologies can identify gene expression patterns over larger areas, with Stereo-seq offering a maximum area of up to 13.2 cm × 13.2 cm.^28^ Furthermore, most of these technologies require only standard sequencing equipment rather than expensive specialized instruments, facilitating their widespread application in ST research. Moreover, sequencing is considerably less time-consuming compared to imaging processes. To date, these technologies have achieved a range of spatial resolutions, spanning from tens of microns to submicron levels. However, their relatively limited sensitivity remains a common limitation. In addition, poly-A-based capture strategies tend to favor the detection of highly-expressed genes and are not compatible with FFPE samples.

## A concise workflow for integrating ST into musculoskeletal system research (Fig. 1)

In the context of musculoskeletal system research, sample compatibility plays a crucial role in the selection of ST platforms. When working with hard tissues such as bone, FFPE samples are often preferred due to the need for decalcification. The compatible sample types for various ST technologies are summarized in Tables 1 and 2. Additionally, previous studies have provided a focused summary of ST platforms specifically adapted for FFPE samples.^72^ The biological questions to be addressed are a prerequisite for selecting an ST platform. Most sequencing-based ST technologies offer the ability to achieve unbiased whole-transcriptome coverage, making them well-suited for hypothesis-generating exploratory studies. In contrast, imaging-based ST technologies provide high-sensitivity detection of target genes, making them more appropriate for hypothesis testing and clinical research.^73^ Furthermore, species compatibility is a crucial consideration in the decision-making process. Imaging-based commercial platforms and probe-based sequencing platforms, such as GeoMx DSP, Visium V2, and Visium HD, are restricted to transcriptome detection in mouse, human, or evolutionarily similar species. Although customization of probe panels is feasible, it substantially increases both the complexity and cost of experiments. After addressing fundamental considerations such as sample compatibility, research objectives, and species adaptability, specific technical parameters, such as sensitivity and spatial resolution, along with the advantages and limitations of each technique, become critical factors for further evaluation. As we have discussed above, trade-offs often exist between various technical parameters within a single technology or platform.^74^ In recent years, advances in ST technologies and the emergence of new commercial platforms have progressed in parallel, both entering a phase of rapid development. Compared to technologies confined to laboratory settings, commercial platforms are more robust and mature, offering essential technical support and user-friendly analytical interfaces, making them a more accessible yet higher-cost option.^74^ Finally, given the complementary nature of imaging-based and sequencing-based ST technologies, employing two complementary methods within the same study, such as the “wide-angle” Visium combined with the “focused” RNAscope,^75^ can significantly enhance our ability to explore the spatial transcriptome landscape, offering a more comprehensive and detailed perspective.

**Fig. 1:**
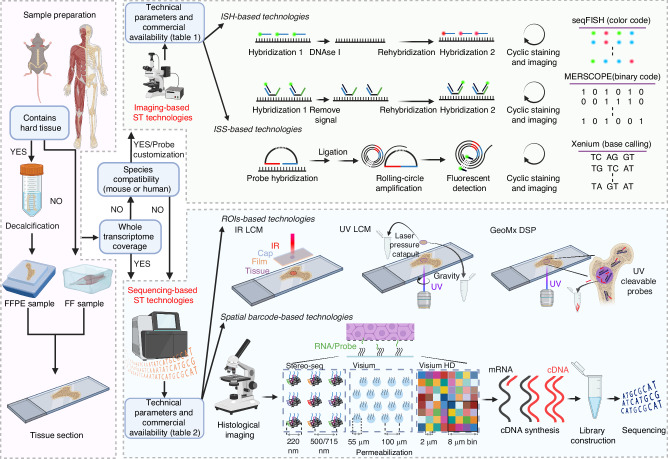
A Concise Workflow for Integrating ST into Musculoskeletal System Research and Overview of the Main ST Technologies. The figure highlights key considerations for conducting ST research in the musculoskeletal system, such as sample types, transcriptome coverage, and species compatibility, in order to select between imaging-based or sequencing-based technologies. It also illustrates the main ST technologies, which researchers can choose based on different technical parameters and commercial availability. However, it is important to acknowledge that the choice of technology is a complex process, and this workflow may not be universally applicable to all biological questions. Researchers should carefully weigh their options when making a technological choice. Furthermore, it should be noted that some probe-based sequencing platforms, such as GeoMx DSP, Visium V2, and Visium HD, are limited to near-whole transcriptome detection in humans and mice only. cDNA complementary DNA. Created in BioRender. Wang, H. (2025) https://BioRender.com/e71m592

## Applications of ST in the musculoskeletal system

Although RNA-seq and scRNA-seq have facilitated our understanding of the physiological and pathological mechanisms within the musculoskeletal system, further clarification is needed regarding the spatial heterogeneity of gene expression, spatial relationships between cells, and their interactions based on spatial context. Currently, the application of ST to the musculoskeletal system is still in its developmental stages. This section aims to introduce the advances in ST concerning the physiological mechanisms of the musculoskeletal system, particularly during developmental processes (Table 3), as well as new findings and breakthroughs enabled by ST in the study of various diseases of the musculoskeletal system (Table 4).

**Table 3:** Advances in the Application of ST to Physiological Mechanisms of the Musculoskeletal System

Author	ST Technologies	Resource	Tissues/ Structures	Sample type	Key research achievements	References
Xiao et al.	Visium/RNAscope	mice	femurs	FFPE	Confirmed the feasibility of applying spatial barcode-based ST technologies to fully mineralized mature long bone tissue. Analyzed the cellular composition and interaction networks of the SSPCs’ microenvironment.	^12^
Zhang et al.	Visium/RNA-ISH	human	embryonic limb	FF	Constructed the first single-cell spatiotemporal transcriptome landscape of human embryonic limb development.	^14^
Mirzazadeh et al.	RRST	mice	growth plate	FF	Identified significantly-upregulated soluble factors in the SOC and SOC-adjacent areas of mice, including *Ccl9*, *Basp1*, and *Apln* in the SOC area and *Msmp* in the SOC-adjacent area which may influence spatially-proximate chondrocytes.	^64^
Piña et al.	Visium/RNAscope	mice	secondary palate	FFPE	Accurately pinpointed the onset of palatal ossification between E14.5 and E15.5, and identified the spatiotemporal localization of marker genes (*Deup1, Lrrc23, Dynlrb2*) during palatal fusion.	^75^
Gribaudo et al.	tomo-seq/RNAscope	organoid models	-	FF	Confirmed that human trunk self-organization organoid model can replicate multi-tissue concomitant morphogenesis of the spinal cord and vertebral column similar to in vivo conditions.	^76^
Chen et al.	Visium/ISS	mice	intervertebral disc	FF	Constructed the first spatial transcriptome atlas of the intervertebral disc.	^77^
Zhang et al.	stereo-seq	mice	shoulder region	not mentioned	Intricately described the complexity and spatial heterogeneity of fibrocartilage attachment site cells in the shoulder region of postnatal mice. Revealed the molecular dynamics during fibrocartilage differentiation.	^78^
Lui et al.	LCM/RNA-ISH	mice	tibial articular cartilage	FF/FFPE	Identified new signaling pathways with spatial regulation during the growth of mouse articular cartilage. Revealed similarities in gene spatial expression patterns from the superficial to the deep regions of joint cartilage and from hypertrophic to resting zones of growth plate cartilage.	^79^
Chau et al.	manual microdissection/RNA-ISH	rat	proximal tibial epiphyses	FF/FFPE	Revealed similarities in gene spatial expression patterns from the superficial to the deep regions of joint cartilage and from hypertrophic to resting zones of growth plate cartilage.	^80^
Bian et al.	Visium/FISH	mice	Hindlimbs	FFPE	Uncovered the critical role of the G protein-coupled receptor ADGRG6 in regulating chondrocyte proliferation and differentiation, as well as maintaining growth plate homeostasis by Indian Hedgehog signaling.	^81^
Tong et al.	LCM	mice	knee joints	FF	Provided critical insights into the molecular and spatial mechanisms driving SOC development. Indicated that mesenchymal progenitors in the periarticular region around epiphyseal cartilage play a critical role in initiating SOC development and forming subchondral bone.	^82^
Tower et al.	Visium	mice	calvaria	FF	Discovered that the presence of sensory innervation maintains the undifferentiated state of mesenchymal cells in cranial sutures to keep cranial sutures patent, while the absence of sensory innervation leads to dysregulation of BMP/TGF-β signaling, manifesting as premature closure of cranial sutures.	^83^
Baccin et al.	LCM	mice	femurs	FF	Established the first spatial atlas of the bone marrow microenvironment.	^84^
D’Ercole et al.	Visium/LCM	mice	tibialis anterior, associated extensor digitorum longus	FF	Examined the distinct morphofunctional regions in muscle and their reaction to reversible nerve injury, emphasizing the polyamine pathway as a possible contributor to muscle atrophy.	^85^
Karlsen et al.	Visium	human	myotendinous junction	FF	Analyzed the different myofibre domains in the human myotendinous junction at the single-nucleus spatial level.	^86^
Steffen et al.	Visium	rat	patellar tendons	FF	Presented the first spatial gene expression landscape of healthy tendon, and clarified the spatial expression patterns of tendon-associated genes.	^87^

**Table 4:** Advances in the application of ST to various diseases of the musculoskeletal system

Author	ST Technologies	Resource	Tissues/ Structures	Sample type	Pathologies/Disorders	References
*Inflammatory Diseases*						
Vickovic et al.	ST	human	synovial tissue	FF	rheumatoid arthritis	^92^
Meng et al.	Visium	human	synovial tissue	FF	rheumatoid arthritis	^93^
Smith et al.	Visium	human	synovial tissue	FF	rheumatoid arthritis	^94^
Rauber et al.	Visium	human	synovial tissue	FF	psoriatic arthritis/rheumatoid arthritis	^95^
Zheng et al.	Visium	human	synovial tissue	FF	osteoarthritis/rheumatoid arthritis	^96^
Carlberg et al.	ST	human	synovial tissue	FF	rheumatoid arthritis/spondyloarthritis	^97^
Hardt et al.	Visium	human	synovial tissue	FF	rheumatoid arthritis	^98^
MacDonald et al.	CosMx SMI	human	synovial tissue	FFPE	rheumatoid arthritis	^99^
Kenney et al.	Visium	mice	popliteal lymph nodes	FF	rheumatoid arthritis	^100^
*Traumatic Diseases*						
McKellar et al.	STRS	mice	tibialis anterior muscles	FF	muscle injuries	^65^
McKellar et al.	Visium	mice	tibialis anterior muscles	FF	muscle injuries	^109^
Young et al.	Visium/smFISH	mice	gastrocnemius muscles/tibialis anterior muscles	FF	muscular dystrophy/muscle injuries	^110^
Larouche et al.	Visium	mice	tibialis anterior muscles	FF	volumetric muscle loss	^111^
Ackerman et al.	Visium	mice	hind paws	FF	tendon injuries	^112^
Cherief et al.	Visium	mice	Achilles tendon	FF	tendon injuries	^113^
Kang et al.	Visium	mice	Achilles tendon	FFPE	traumatic heterotopic ossification	^114^
Tower et al.	Visium	mice	digit	FF	limb defects	^117^
Wan et al.	Visium	mice	calvarium	FF	bone defects	^118^
Jiang et al.	Visium	mice	femurs	FFPE	normal and pathological fractures	^119^
Rios et al.	Visium	mice/human	pseudarthrosis	FFPE	fracture in neurofibromatosis type 1	^120^
Mathavan et al.	Visium	mice	femurs	FFPE	fractures	^121^
Foster et al.	Visium	mice	dorsal skin	FF	skin injuries	^123^
Yang et al.	Visium	mice	dorsal skin	FF	skin injuries	^124^
Chen et al.	Visium	human	umbilical cord	FF	skin injuries	^125^
*Degenerative Diseases*						
Fan et al.	Geo-seq	human	knee articular cartilage	FF	osteoarthritis	^134^
Yang et al.	Visium	human	anterior cruciate ligaments	not mentioned	osteoarthritis	^137^
Perez et al.	GeoMx DSP	human	vastus lateralis	FFPE	sarcopenia	^138^
Akbar et al.	Visium	human	hamstring tendon/supraspinatus tendon	not mentioned	tendinopathy	^139^
Fu et al.	Visium	human	supraspinatus tendon	not mentioned	tendinopathy	^140^
*Tumorous Diseases*						
Zhang et al.	Visium	human	tumor specimens	FF	chordoma	^148^
Wrenn et al.	GeoMx DSP	mice	tumor specimens	FFPE	Ewing sarcoma	^150^
Li et al.^a^	ST	human	tumor specimens	FF	pan-cancer with M1 macrophage infiltration	^152^
Ihle et al.	GeoMx DSP	human	tumor specimens	FFPE	prostate cancer bone metastasis	^156^

^a^Li et al. did not perform ST experiments and their ST data were sourced from SpatialDB.^230^

### Advances in the application of ST to physiological mechanisms of the musculoskeletal system

**Fig. 2:**
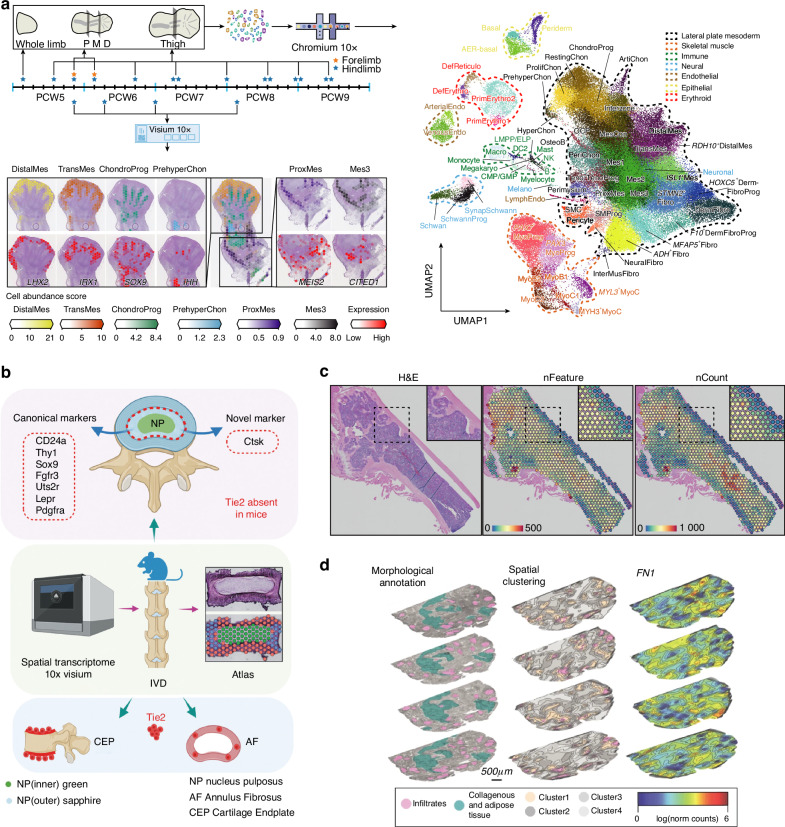
Application of ST in Physiological Mechanisms and Inflammatory Diseases of the Musculoskeletal System. **a** At different time-points of human embryonic limb development, scRNA-seq and ST have been used to construct a spatiotemporal transcriptomic map. UMAP visualization of 125 955 human embryonic limb cells. In human posterior limb tissue slices at PCW6.2, spatial heatmaps of specific cell types and corresponding marker genes.^14^ *Copyright* © *2023, The Author(s)*. **b** Multiple canonical markers detected in the murine NP region, but Tie2 only in the CEP or AF region.^77^ © *2024 The Authors. Advanced Science published by Wiley-VCH GmbH*. **c** H&E-stained sections of adult murine femur and heatmaps of the number of unique genes (nFeature) or unique transcripts (nCount) detected at each capture site.^12^ *Copyright* © *2023, The Author(s)*. **d** Morphological annotation, spatial clustering, and spatial expression pattern of *FN1* of a serum-positive RA patient sample.^92^ *Copyright* © *2022, The Author(s)*. P proximal, M middle, D distal, PCW post-conception week, infiltrates leukocyte infiltration sites

ST provides new insights into the physiological mechanisms of the musculoskeletal system at various developmental stages including the embryonic, juvenile, and mature phases. The embryonic development stage of the musculoskeletal system involves a complex and finely-coordinated evolution of cells and changes in gene spatial expression patterns. Although studies on model organisms have elucidated the fundamental mechanisms of limb development in vertebrates, the spatiotemporal characteristics of this process in humans remain incompletely understood. Zhang et al.^14^ conducted an analysis of multiple time points during human embryonic limb development using scRNA-seq and Visium. Utilizing Uniform Manifold Approximation and Projection (UMAP), they visualized 125 955 human embryonic limb cells identified through scRNA-seq. The spatial data revealed the spatial distribution of specific cell types and the spatial expression patterns of their corresponding marker genes (Fig. 2a). Consequently Zhang et al.^14^ successfully provided the first intricate depiction of the single-cell spatiotemporal transcriptome landscape of human embryonic limb development. The study further deciphered the cellular evolutionary pathways and cell spatial positioning determination processes from early limb formation to complete morphogenesis. It revealed two phases of human skeletal muscle development characterized by different cellular states and identified temporally-regulated gene expression patterns crucial for limb formation. The detailed portrayal of human limb development by Zhang et al.^14^ is valuable for gaining a deeper understanding of the mechanisms behind congenital limb syndromes and improving their diagnosis and treatment strategies. Moreover, this study also revealed substantial homology between mice and humans, demonstrating high similarity in the spatial expression patterns of genes controlling forelimb/hindlimb and proximal–distal identity, confirming mice as a reliable model for studying human physiological and pathological mechanisms.^14^ In addition, ST also contributed to the spatial analysis of the palatal fusion process^75^ and to the development and validation of complex higher-order organoid models related to embryonic development.^76^

The juvenile phase of the musculoskeletal system represents a dynamic period of growth and differentiation, marked by significant cellular turnover and the establishment of mature tissue structures. ST enables precise parsing of gene spatial expression patterns during this critical developmental window, providing unique insights into cellular hierarchy and lineage specification through the combination of lineage tracing. Nucleus pulposus progenitor cells (NPPCs) play a crucial role in maintaining cellular refreshment and supporting the development of nucleus pulposus (NP) tissue.^77^ However, the spatial differentiation trajectory of NP cells remains to be further explored. Chen et al.^77^ constructed the first spatial transcriptome atlas of the intervertebral disc (IVD) of juvenile mice using Visium, combined with lineage tracing to identify cells located at the periphery of the NP and expressing Cathepsin K (Ctsk) as NPPCs, which generate the entire NP adult tissue. Meanwhile, Tie2, long suggested as a marker for NPPCs, was not observed in the juvenile mouse NP but was found in the cartilage endplate (CEP) and annulus fibrosus (AF), as confirmed through ISS (Fig. 2b). Spatial analysis of the IVD suggests that previous reports regarding Tie2^+^ cells in NP tissue may have been influenced by potential contamination from Tie2^+^ cells in adjacent tissues such as the CEP or AF. Moreover, the existence of Tie2^+^ NPPCs in humans and other species remains to be further investigated. Beyond the IVD, ST has also been applied to the analysis of developing fibrocartilage attachment sites,^78^ articular cartilage,^79^ growth plate cartilage,^80,81^ the secondary ossification center (SOC),^64,82^ and the cranium.^83^

Analyzing the mature stage of the musculoskeletal system through ST helps improve understanding of its physiological characteristics under steady-state conditions and provides a foundation for exploring various diseases. The homeostatic microenvironment of bone marrow provides essential support for the physiological self-renewal and differentiation of SSPCs.^12^ Baccin et al.,^84^ using scRNA-seq and LCM-seq, established the first spatial atlas of the bone marrow microenvironment, revealing different perivascular niches contributed by the spatial heterogeneity of Cxcl12-abundant reticular cell subpopulations. Xiao et al.^12^ confirmed the feasibility of applying spatial barcode-based ST technologies to fully mineralized adult long bones. Specifically, the study utilized the Visium platform with a probe panel covering approximately 20 000 genes to analyze decalcified FFPE sections of mature mouse femur, successfully demonstrating a high transcript recovery efficiency (Fig. 2c). Xiao et al.^12^ further analyzed the cellular composition and interaction networks within the microenvironment of the SSPCs, providing a comprehensive understanding of both local and global regulatory SSPC networks. Additionally, ST has been applied to the spatial characterization of mature skeletal muscle,^85^ myotendinous junctions,^86^ and tendons.^87^

### Applications of ST in inflammatory diseases of the musculoskeletal system

Rheumatoid arthritis (RA) is a common chronic systemic autoimmune disease affecting between 0.25% and 1% of the global population.^88,89^ Despite the continuous development and application of disease-modifying antirheumatic drugs, which control disease activity in most patients with RA, a proportion of patients still experience limited treatment benefits.^90^ The pathological mechanisms behind the clinical manifestations and treatment responses of RA remain to be explored further.^91^ ST offers a new means to reach an in-depth understanding of the complex pathological mechanisms of musculoskeletal inflammatory diseases such as RA. Vickovic et al.^92^ analyzed the differences in cellular composition and spatial organization and interactions of cells in the synovial sites of patients with seropositive and seronegative RA, constructing an exploratory multi-dimensional view of RA by combining tissue morphology with spatial transcriptome landscapes. To further explore the pathological mechanisms of RA and discover new therapeutic target clues, researchers have conducted detailed studies on fibroblast-like synoviocytes (FLSs),^93–96^ immune cells,^92,97–99^ and synovial draining lymph nodes^100^ using ST.

FLSs are considered tissue-resident cells involved in the initiation, maintenance, and resolution of chronic inflammation in arthritis, and their activation is a key step in the occurrence and development of arthritis.^101,102^ Smith et al.^94^ by analyzing the chromatin accessibility and gene expression profiles of FLSs, constructed a spatial atlas of FLS cell states influenced by local exposure to TNF, IFN-γ, and IL-1β during active RA. It is particularly noteworthy that inhibition of IL-1 may improve the activated state of the lining FLSs and prevent further joint damage.^94^ The role of FLSs during the remission and relapse of inflammation remains a topic for further investigation. Rauber et al.^95^ found that during the remission process of arthritis, FLSs transitioned from an MMP3 ^+^ /IL6^+^ phenotype to a CD200 ^+^ DKK3^+^ phenotype. Their ST analysis of synovium from RA and psoriatic arthritis patients showed that MMP3 ^+^ /IL6^+^ FLSs were spatially proximate to inflammatory immune cells in areas of active inflammation, whereas CD200 ^+^ DKK3^+^ FLSs co-localized with type 2 innate lymphoid cells in areas of inflammation resolution, further indicating that CD200^+^ FLSs formed an inflammatory resolution-promoting microenvironment in arthritis.^95^ Meng et al.^93^ integrated single-cell and spatial transcriptomics data to construct the spatial transcriptome landscape of the synovium in patients with RA undergoing sustained remission and relapse, identifying co-localization in the lining layer of recurrent RA patients among macrophages considered precursors to osteoclasts that express CTSK^+^, pro-inflammatory M1-type macrophages, and CD55^+^ lining FLSs, which significantly increased in proportion in the synovium during relapse. The study by Meng et al. also revealed the crucial role of the fibroblast growth factor (FGF) signaling pathway in the recurrence of RA, with a notable increase in expression levels in the lining FLSs of recurrent RA patients.^93^ Further research indicated that knocking out FGF10 or inhibiting FGF receptor 1 (FGFR1) effectively regulated the activity of the FGF pathway, reducing bone erosion, and offering new hope for the remission of recurrent RA.^93^

Immune cells play an undeniable role in the occurrence and progression of arthritis. In chronic inflammatory sites of non-lymphoid tissues, organized structures formed by the aggregation of immune cells are referred to as tertiary lymphoid organs (TLOs).^103^ Although scRNA-seq provides unique data on the cellular heterogeneity of RA synovial tissue,^104,105^ the limited understanding of the spatial organization of TLOs within RA synovium hinders our deeper insights into the pathogenesis of RA and its therapeutic responses. Vickovic et al.^92^ conducted serial sectioning of synovial tissue from RA patients, identifying leukocyte infiltration sites through morphological annotation, and delineating key spatial regions characterized by distinct gene expression patterns using unsupervised clustering approaches. The study also demonstrated the spatial expression pattern of *Fibronectin 1 (FN1)*. The expression level of *FN1* is positively correlated with transforming growth factor beta (TGF-β) activity, and TGF-β, due to its pivotal role in joint destruction, is considered a crucial target for monitoring disease progression and developing therapeutic strategies.^106^ In summary, Vickovic et al. constructed a fused morphological 3D spatial transcriptome landscape of synovial tissue from RA patients (Fig. 2d). The study also identified differences in the spatial gene expression patterns between seropositive and seronegative RA patients within or around TLOs. By integrating scRNA-seq data, it further revealed the specific localization patterns of different cell types.^92^ Additionally, other ST studies have contributed to uncovering the key roles of T cells,^97^ B cells,^98^ and dendritic cells^99^ in RA synovium.

Kenney et al.^100^ were the first to use combined single-cell transcriptomics and ST to analyze the pathological changes in draining lymph nodes during the progression of RA. The study revealed that in the draining lymph nodes of late-stage arthritis, close crosstalk between ALCAM^+^ macrophages and CD6 ^+^ T cells promoted B cell differentiation into plasma cells and IgG2b^+^ class switching.^100^ The aggregation of IgG2b^+^ plasma cells near the MARCO^+^ sinusoids in the draining lymph nodes was associated with the exacerbation of late-stage arthritis.^100^ The findings of Kenney et al. further deepen our understanding of the relationship between arthritis progression and lymphatic dysfunction. In future, the therapeutic potential of targeted inhibition of CD6 in RA still requires further exploration.

### Applications of ST in traumatic diseases of the musculoskeletal system

Globally, trauma accounts for over one in ten deaths, with non-fatal injuries potentially leading to impaired motor function and reduced quality of life.^107^ Clinicians and researchers have long pursued functional regeneration and the avoidance of non-functional fibrotic repair in tissues such as skin, muscle, and tendon following trauma, as well as deciphering the spatial molecular mechanisms linking local mechanical environments to cellular responses during fracture healing to promote optimal bone repair. However, limited understanding of the cellular and molecular spatiotemporal mechanisms during healing has prevented the consensus on effective improvements in healing outcomes. ST offers new hope for studying regenerative mechanisms and identifying intervention targets in skeletal muscle, tendon and bone, as well as skin.

**Fig. 3:**
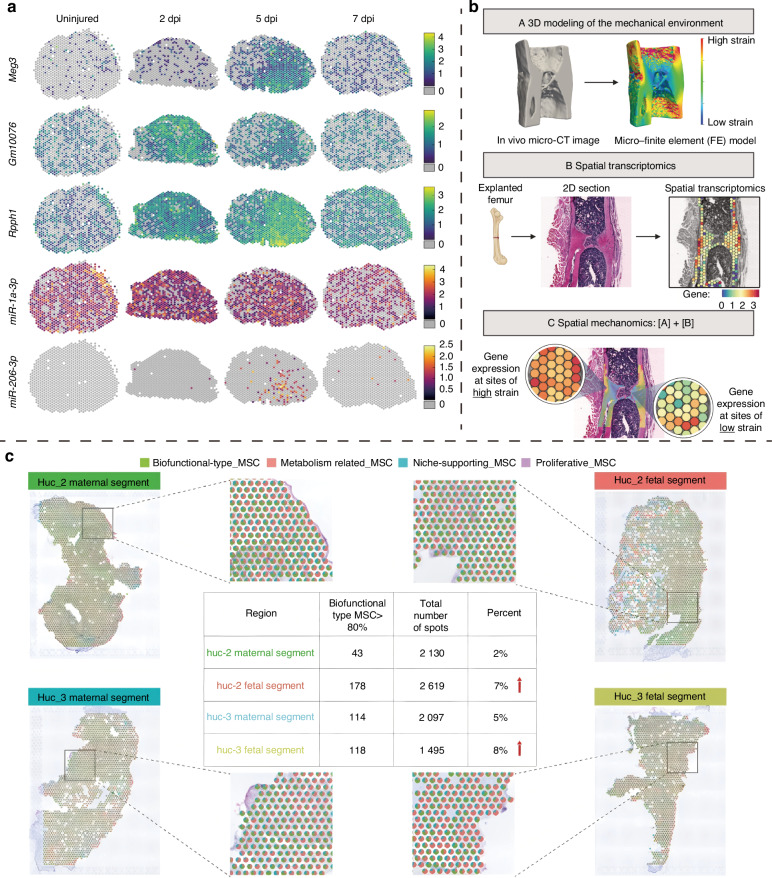
Application of ST in Traumatic Diseases of the Musculoskeletal System. **a** Spatiotemporal expression pattern of *Meg3*, *Gm10076*, *Rpph1*, *miR* − *206* − *3p* and *miR* − *1a* − *3p* at different time-points before and after injury during the regeneration process of murine anterior tibial muscle.^65^ *Copyright © 2022, The Author(s)*. **b** In vivo micro-CT imaging is used for micro-finite element analysis to create 3D landscapes of the mechanical environment at the tissue scale. ST analysis of explanted femurs was performed to construct 2D spatial transcriptome landscapes. Finally, gene spatial expression patterns under different mechanical strains were constructed through visual alignment.^121^ *Copyright © 2025, The American Association for the Advancement of Science*. **c** Deconvolution of Visium capture spots from different regions of the umbilical cord was performed to visualize cell type proportions. The color coding represents different cell types, while pie charts illustrate the proportion of each cell type at each spot. The proportion of spots in the fetal segment of the umbilical cord, where functional MSCs comprise over 80% of the cells, was higher compared to the maternal segment.^125^*© 2022 The Authors. Advanced Science published by Wiley-VCH GmbH*. dpi day post-injury, Huc human umbilical cord

Muscle and tendon injury repair is a coordinated process involving multiple cell types and complex gene regulatory networks.^65,108^ ST has been used to analyze the spatial characteristics of cellular and gene expression patterns in skeletal muscle regeneration,^65,109–111^ tendon healing,^112,113^ and traumatic heterotopic ossification.^114^ As an example, McKellar et al.^65^ applied STRS to the skeletal muscle regeneration process, conducting ST analysis on uninjured tibialis anterior muscles and those at days 2, 5 and 7 post-injury in mice. They robustly identified previously undetected or poorly-detected transcripts, including *Meg 3*, *GM 10076*, *Rpph1*, as well as highly-abundant mature miRNAs, such as *miR* − *206* − *3p* and *miR* − *1a* − *3p*, and demonstrated their corresponding spatiotemporal expression patterns (Fig. 3a). While the pivotal role of miRNAs in skeletal muscle regeneration has been extensively studied,^115,116^ the standard Poly-A-based Visium workflow has limited their detection in a spatial context. McKellar et al. made a pioneering contribution to the spatial transcriptome landscape of skeletal muscle regeneration by including non-coding RNA components.^65^ As another instance of ST being applied to muscle regeneration, Larouche et al.^111^ attempted to elucidate the cellular and molecular spatiotemporal mechanisms behind failed muscle tissue regeneration and fibrotic scar formation following volumetric muscle loss, mediated by immune and stem cell dysregulation. In tissue sections, the volumetric muscle loss area can be divided into a defect zone with complete muscle loss, an intact zone with fully preserved muscle, and a transition zone in between.^111^ Analyses showed that at day 7 post-injury, scar-associated macrophages colocalized with mesenchymal-derived cells in the defect zone, characterized by high expression of inflammation-and collagen deposition-related genes, while muscle stem cells were primarily found in the transition zone, enriched with developmental myogenic genes, and were almost absent from the defect zone.^111^ Over time, inflammation in the volumetric muscle loss defect and transition zones subsided, but fibrotic remodeling intensified.^111^ Further analysis indicated that interactions between scar-associated macrophages and mesenchymal-derived cells promoted fibrosis progression, which was unfavorable for MuSC-mediated regeneration, and that disrupting this crosstalk via TGF-β inhibition created a microenvironment conducive to MuSC-mediated muscle regeneration which also impeded fibrotic remodeling in the defect zone.^111^

Up to now, ST has been applied in studies related to digit regeneration,^117^ cranial defect repair mediated by bone repair materials,^118^ and both normal and pathological fracture healing.^119–121^ Given the important role of mechanoregulation in skeletal regeneration and reconstruction, Mathavan et al.^121^ developed an ST-based mechanomics platform to explore the transcriptomic features of cells in different local mechanical environments during fracture healing within the spatial framework. Specifically, the researchers first performed in vivo micro-CT imaging on a mouse femoral defect model weekly and further used micro-finite element analysis to construct a 3D model of the mechanical environment. Three weeks after the fracture, the mice were divided into two groups: the Loaded group, which received cyclic mechanical loading, and the Control group, which received sham loading. Subsequently, the researchers conducted ST analysis on longitudinal sections of the femurs from both groups at five weeks post-fracture to construct a spatial transcriptomic landscape. Finally, by integrating spatial multimodal data, including bone morphology, mechanical environment, and gene spatial expression patterns, the study provided a clearer understanding of the molecular mechanisms of fracture healing under local mechanical regulation (Fig. 3b). The study indicates that gene expression features in high-strain regions are associated with osteogenic responses, characterized by upregulation of genes such as *Spp1* and *Col1a2*. In contrast, gene expression features in low-strain regions show dominance of bone resorption, with upregulation of genes like *S100a8* and *Ncf1* (ref. ^121^). In addition, spatial analysis of differentially-expressed genes at the fracture site confirmed a significantly enhanced osteogenic response in the Loaded groups compared to the Control group, as evidenced by the upregulation of osteogenic differentiation and activity markers (e.g., *Sp7*, *Bglap*), mineralizing osteocyte markers (e.g., *Phex*, *Dmp1*), and mature osteocyte markers (e.g., *Mepe*, *Sost*).^121^ In the future, utilizing ST technologies with higher resolution and constructing 3D transcriptomic landscapes to build a 3D mechanomics platform will offer finer and more intuitive insights in this field, promoting the discovery of mechano-responsive targets to facilitate fracture healing.

The wound healing process involves a complex and finely-coordinated cellular and molecular mechanism, and research in this area has been ongoing for over a century.^122^ ST has provided valuable spatial insights into both the normal process and the involvement of biomaterials during wound healing.^123,124^ Furthermore, ST has contributed to advances in cell-based therapies related to wound repair.^125^ Mesenchymal stem cells (MSCs), for which Wharton’s jelly MSCs (WJ-MSCs) make an excellent representative model, have broad clinical application potential for promoting tissue repair and regeneration^126^ and regulating immunity.^127^ However, their high heterogeneity may be one of the key reasons for their inconsistent clinical efficacy.^128^ Chen et al.^125^ conducted a systematic analysis of WJ-MSCs by combining scRNA-seq and ST. scRNA-seq identified four subtypes of WJ-MSCs, with the S100A9 ^+^ CD29 ^+^ CD142^+^ functional MSC subtype, which promotes wound healing, showing potential as a therapeutic agent for wound healing. ST analysis of maternal and fetal segments from two human umbilical cords (UCs) revealed the spatial heterogeneity of molecular and functional characteristics across different regions of the UC. Finally, the researchers used SPOTlight^129^ to integrate scRNA-seq and ST data for deconvolution of Visium capture spots. By calculating the proportion of capture spots in which functional MSCs constituted more than 80% of the cells, they found that functional MSCs were relatively enriched in the fetal segment of the UC compared to the maternal segment^125^ (Fig. 3c). This suggests that the fetal segment of the UC is an ideal source of this MSC subtype.

### Applications of ST in degenerative diseases of the musculoskeletal system

Osteoarthritis (OA) is a common degenerative disease primarily affecting the knee and hip joints, characterized by joint pain and functional impairment.^130^ In South Korea, the prevalence of knee OA among individuals over 50 years old is as high as 35.1% (ref. ^131^), and the prevalence of OA continues to rise globally.^132^ Differences in subtype among OA patients and the spatial heterogeneity of chondrocytes present challenges for the precise diagnosis and treatment of OA.^133^ Fan et al.^134^ integrated Geo-seq and scRNA-seq data to create a single-cell and regional spatially-resolved transcriptome landscape of human knee cartilage, both with and without OA, identifying 11 chondrocyte clusters and their specific marker genes, including newly-identified pre-inflammatory and inflammatory chondrocyte clusters. As a benefit of the acquisition of spatial information, Fan et al.^134^ discovered that in OA, most chondrocyte clusters are situated in transcriptionally-quiescent middle and deep zones, whereas prehypertrophic chondrocytes and prefibrocartilage chondrocytes are primarily concentrated in areas of the joint surface and superficial regions that are transcriptionally active and enriched with OA-associated differentially-expressed genes. Further studies indicated that the inflammatory chondrocyte group has the potential to activate MIF-CD74-mediated cartilage degradation in the knee joints of OA patients, that the prehypertrophic chondrocytes and prefibrocartilage chondrocytes groups are crucial for distinguishing individuals with or without OA and for OA subtyping respectively, and that the hypertrophic chondrocyte group is a key cluster associated with susceptibility to OA in European and East Asian populations, providing important clues for precise diagnosis and interventions for OA.^134^

The anterior cruciate ligament is a crucial ligament of the knee joint, and its degeneration can lead to knee instability and chronic pain, potentially promoting the development and progression of OA.^135,136^ Yang et al.^137^ combined single-cell transcriptomics and ST to map the single-cell spatial transcriptome landscape of healthy and degenerated anterior cruciate ligaments, identifying and localizing various cell subtypes. By analyzing their interactions, Yang et al.^137^ found that FGF and TGF-β signaling pathways might mediate extracellular matrix remodeling in anterior cruciate ligament degeneration, and targeting FGF7–FGFR1 and TGFB1–TGFBR2 could be effective therapeutic strategies. The proximity of fibroblasts to immune and endothelial cells, revealed by ST, further supports the analysis of cell interactions.^137^

ST has also been applied to the study of aging skeletal muscle^138^ and tendon.^139,140^ Tendinopathy, also a common degenerative disease of the musculoskeletal system, is accompanied by pain and causes impaired motor function, severely affecting quality of life. The incidence of rotator cuff tendinopathy is as high as 5.5% (refs. ^141,142^). The unclear molecular and cellular mechanisms underlying tendinopathy have led to the lack of a unified and effective treatment method. Through combined single-cell and spatial transcriptomics analysis of healthy tendons and tendon samples from patients with tendinopathy, Akbar et al.^139^ revealed dysregulation of immune homeostasis in tendons with tendinopathy initiated by endothelial and matrix cells. Fu et al.^140^ identified and located tendon cell subgroups, further proposing that targeting spatially-proximate endothelial cell subgroups and macrophages, which alter the niche of tendon stem/progenitor cells, might be effective therapeutic strategies. They also discovered the developmental characteristics of tendinopathy from inflammatory infiltration through chondrogenesis to chondral ossification, suggesting that the essence of tendinopathy is heterotopic ossification of the tendon.^140^ Future research that distinguishes the severity of tendinopathy rather than simply categorizing samples as healthy or diseased may provide a more nuanced perspective.

### Applications of ST in tumorous diseases of the musculoskeletal system

The tumor microenvironment (TME) is composed of dynamically-changing populations of various cell types and extracellular matrix, and analyzing its complex cellular heterogeneity and intercellular interaction networks in a spatial context provides new insights into the mechanisms of tumor occurrence and progression, prognosis assessment, and the development of new tumor markers and therapeutic targets.^143^ Cancer-associated fibroblasts (CAFs) are one of the predominant stromal cell types in the TME and play a pivotal role.^144,145^ Chordomas, originating from embryonic remnants of the notochord, are rare mesenchymal tumors most commonly found in the clival region and sacrococcygeal area.^146,147^ Zhang et al.^148^ used ST to confirm the presence of a novel CAF cluster, termed endoplasmic reticulum stress-CAFs (ERS-CAFs), in the chordoma TME, originally identified via scRNA-seq. The spatial data indicated that ERS-CAFs are located close to tumor cells, potentially promoting tumor progression through direct crosstalk, and the proximity of ERS-CAFs to tumor cells correlates with the severity of malignancy and patient prognosis.^148^ Ewing’s sarcoma is a poorly-differentiated malignancy of small round cells, occurring predominantly during adolescence, and has a poor prognosis.^149^ Wrenn et al.^150^ combined ST and single-cell proteomics to identify spatial heterogeneity among Ewing’s sarcoma cell subpopulations. Their study, augmented by multiplex immunofluorescence staining, found CD73^+^ Ewing’s sarcoma cells exhibited similarities with CAFs in upregulating extracellular matrix protein expression and deposition, and they, therefore, categorized this subgroup as CAF-like tumor cells.^150^ Their findings underscore the significant role of CAF-like tumor cells in remodeling the TME to promote tumor initiation and progression.^150^

Beyond CAFs and tumor cells, immune cells in the TME also play a crucial role in either promoting or inhibiting tumor initiation and development, drawing significant research interest.^151^ Integrating data from bulk, single-cell, and spatial transcriptomics, validated through immunostaining, established proteasome activator complex subunit 2 (PSME2) as a pan-cancer biomarker in cancers infiltrated by M1 macrophages.^152^ Osteosarcoma is a highly-aggressive malignant bone tumor primarily affecting children with a low survival rate.^153^ Researchers found that overexpression of PSME2 in osteosarcoma cells significantly inhibits their proliferation, invasion, and migration, and consequently they screened the PSME2 agonist Irinotecan, which in combination with paclitaxel, was found to synergistically promote apoptosis in osteosarcoma cells.^152^

Metastatic prostate cancer has a high mortality rate, with bones being a primary metastatic site.^154^ Bone metastases from prostate cancer exhibit three pathological subtypes: lytic, blastic, and mixed.^155^ Ihle et al.^156^ used GeoMx DSP and immunohistochemical staining to reveal differences in immune cell-enriched biological pathways between blastic and lytic lesions in the TME. The former displayed high levels of pSTAT3 and components of the JAK-STAT pathway, while the latter showed enrichment of pAKT activity and PI3K-AKT pathway components. Compared to lytic lesions, blastic lesions have multiple enriched immune checkpoints, including IDO-1, OX40L, B7-H4, and PD-L1, highlighting potential targets of immune therapy in bone metastases of blastic prostate cancer.^156^

ST holds substantial potential in analyzing the TME of musculoskeletal systems, providing deep insights into tumor pathology and offering new perspectives for disease diagnosis, treatment, and prognostic prediction. Furthermore, ST aids in validating regulatory networks constructed based on bioinformatics analysis from RNA-seq data. For instance, Huang et al.^157^ developed a specific regulatory network based on prognostic stemness-related signatures for infiltrative breast cancer bone metastases, supported by ST and other multi-omics data.

## Challenges and prospects (Fig. 4)

### Challenges in the application of ST to the musculoskeletal system

In current single-cell and spatial multi-omics research, most high-quality data originate from soft tissue samples. Related tissues in the musculoskeletal system, such as bone, cartilage, and tendon, contain tougher, denser, and even mineralized extracellular matrices, with relatively lower cell content,^158^ presenting challenges for conducting single-cell and spatial multi-omics studies. This section discusses the challenges and potential solutions in sample preparation for ST, the permeabilization process, and effective identification of rare cell types in the bone marrow, with the aim of promoting wider application of ST to the musculoskeletal system.

**Fig. 4:**
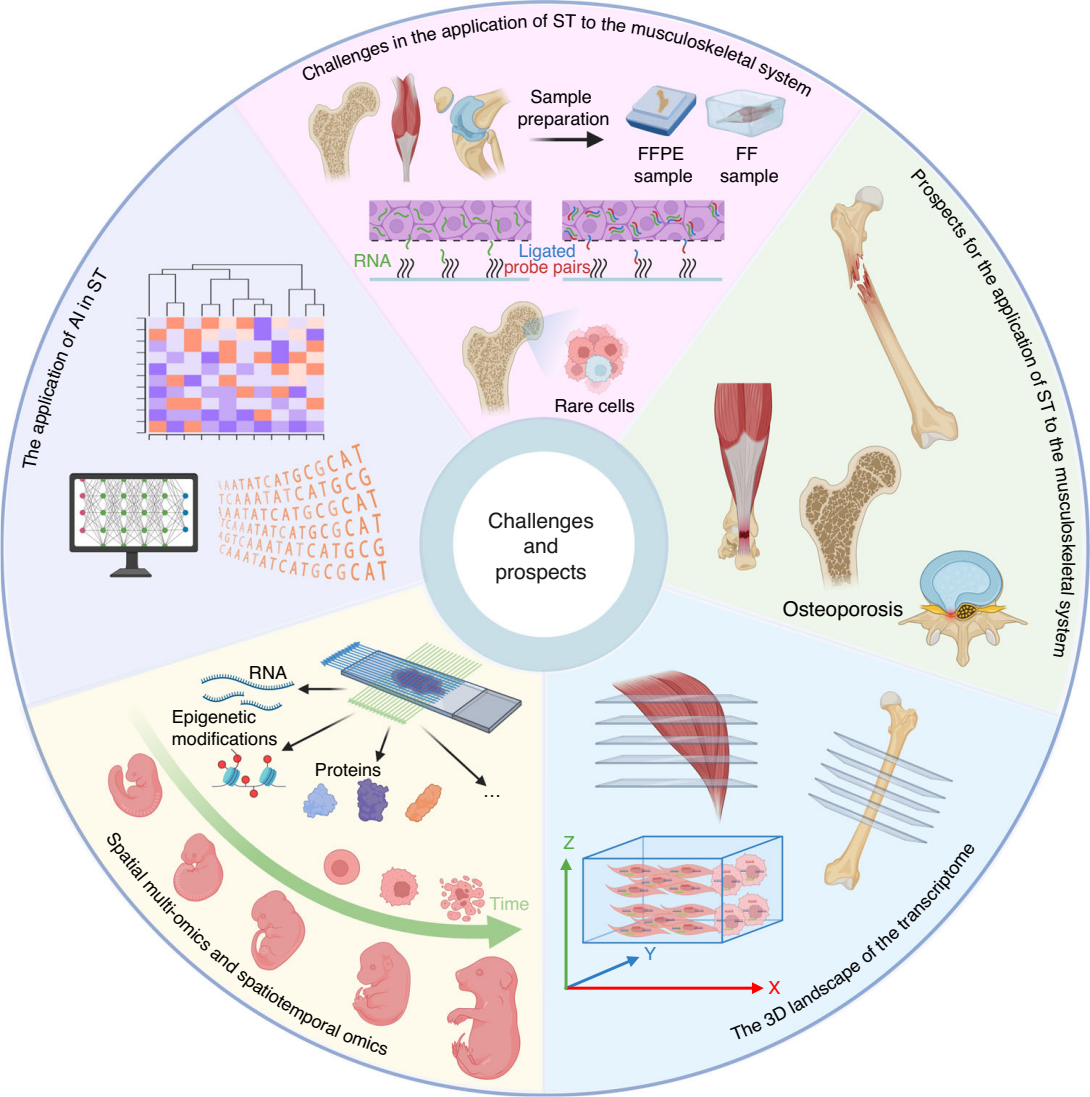
Challenges and Prospects. Challenges and prospects faced by spatial transcriptomics itself and its application to the musculoskeletal system. AI Artificial intelligence, FFPE Formalin fixed and paraffin embedded, FF Fresh frozen. Created in BioRender. Wang, H. (2025) https://BioRender.com/e71m592

Due to the dense and hardened nature of bone and sclerotic bone lesions, an additional decalcification step is required in sample processing compared to soft tissues.^159^ However, most decalcification agents contain strong acids that degrade nucleic acids, leading to RNA degradation.^160^ In contrast, using EDTA for decalcifying bone specimens significantly improves nucleic acid recovery rates, and ultrasonic vibration can reduce decalcification time.^161^ In addition, performing daily imaging assessments during the decalcification process allows for determination of the minimum decalcification time required for specific samples, thereby mitigating the negative impact of this process on RNA quality.^120^ Standardized, nucleic acid-friendly mild decalcification protocols still need further development and improvement. The extracellular matrix of cartilage tissue is primarily composed of proteoglycans and collagen, with cellular content making up only about 5%–10% of the total volume.^162^ The low cellularity of cartilage means that the total RNA content is likely to be lower than that of tissues with higher cell densities, highlighting the importance of maintaining adequate quantities and quality of RNA when conducting transcriptomic analyses on cartilage tissues. During the preparation of cartilage samples, rapidly processing samples at low temperatures to inactivate RNAses, avoiding the use of chemical fixatives, choosing fresh samples and minimizing storage time, and quickly thawing frozen samples are widely agreed-upon methods to effectively prevent RNA degradation.^158^ Notably, it has been reported that RNA degradation levels are significantly increased in cartilage samples from arthritis patients.^163^ Special attention should be given to RNA preservation when studying such samples. For tendon tissue, due to its dense and parallel arrangement of collagen fibers and relatively thin structure, obtaining high-quality longitudinal sections of tendons also presents certain challenges.^87^ Notably, a comprehensive protocol for preparing ST samples from FFPE tissues of the murine musculoskeletal system, including bone and muscle, has recently been established. This development is poised to facilitate the broader application of ST in musculoskeletal system research.^164^

FFPE is a widely-used biological sample fixation method in clinical practice, offering significant advantages, including long-term preservation of tissue samples at room temperature while maintaining structural and morphological integrity.^165^ Importantly, FFPE tissues adhere better to slides than FF samples, reducing the likelihood of tissue detachment. However, the FFPE protocol also has limitations; excessive fixation can cause extensive RNA cross-linking, reducing RNA quality.^165^ A de-crosslinking step can improve RNA accessibility and integrity and thus enhance analytical quality. However, researchers must find a fine balance between adequate de-crosslinking and preventing RNA degradation during this process. Xiao et al.^12^ and Ihle et al.^156^ have confirmed the feasibility of applying ST to FFPE samples of normal bone and of prostate cancer bone metastases, respectively. Currently, many ST technologies have been adapted for FFPE samples, providing a platform for ST analysis of highly-mineralized tissues and making ST more accessible for clinical research. Since FF tissues avoid RNA cross-linking and degradation due to long-term storage, their gene detection efficiency is superior to that of FFPE tissues.^62,68^ To obtain high-quality ST data, analyzing FF tissues remains an indispensable choice. The technique of RRST, which has the capability to analyze FF samples with low-quality RNA, offers three improvements over the commercially-available Visium FFPE solution: it uses a shorter formalin fixation step and omits the de-crosslinking step to prevent RNA cross-linking and degradation, and it includes a baking step to enhance tissue section adhesion and thus address the propensity of FF tissues to detach.^64^ RRST has been successfully applied to FF samples of mouse bone and cartilage tissues.^64^ In the future, combining this improved strategy with higher spatial resolution, spatial barcode-based ST technologies will enable researchers to gain more refined analytical perspectives from decalcified hard tissues and other FF samples with low RNA quality.

Currently, two main permeabilization strategies are generally applied. The first is the PolyA capture-based strategy used in Visium V1, where mRNA in the tissue is released and captured by oligonucleotide sequences containing Poly dT on the capture panel. The second is the probe-based strategy employed in Visium V2 and Visium HD, which uses paired probes targeting the protein-coding regions of RNA based on a gene panel.^64^ Subsequently, ligated probe pairs, rather than RNA, are released from the tissue and captured by the capture panel. The preferred tissue samples for the PolyA capture-based strategy are FF samples, as they preserve polyadenylated transcripts well. The duration of permeabilization for the PolyA capture-based strategy needs to be experimentally determined to achieve the strongest fluorescence signal and minimal lateral diffusion of RNA. Optimizing permeabilization time is a delicate balancing act since it is necessary to sufficiently capture RNA while avoiding prolonged permeabilization that could cause lateral RNA diffusion. However, the optimal permeabilization time may vary between different tissues, and when a section contains different tissues such as bone, cartilage, and soft tissues like muscle, researchers need to select the optimal permeabilization time for the specific tissue area to be focused on, or consider the whole section to balance the suboptimal permeabilization time suitable for all tissues. There are no reported strategies that allow different degrees of permeabilization on the same section for different tissues, limiting the use of the PolyA capture-based strategy on sections containing multiple tissue types. Due to the degradation and fragmentation of RNA molecules in FFPE samples,^166^ the capture efficiency of the PolyA capture-based strategy significantly decreases. Therefore, for FFPE samples, researchers need to choose a probe-based strategy which uses a uniform permeabilization scheme for different tissues without the need to experiment with permeabilization times. However, it is important to note that, as previously mentioned, probe-based strategies are limited to humans, mice, or evolutionarily similar species and are not suitable for other models, such as axolotls.^167^ RRST, compared to the standard Visium probe-based strategy, significantly increases the number of unique genes and unique molecular identifiers detected in cartilage tissue, with a more uniform distribution, producing high-quality bone and cartilage ST data for analysis.^64^ The dense and extensively cross-linked extracellular matrix of cartilage and tendon may be a potential obstacle for transcript or probe permeabilization. However, there have been no reports demonstrating enhanced permeabilization effects by enzymatic degradation of the extracellular matrix during cartilage and tendon permeabilization, and its effectiveness and potential impacts on transcriptional profile and spatial accuracy require further consideration.

Currently, ST studies related to cartilage tissue and its sample preparation are relatively limited. Cartilage is an essential component of the intervertebral disc, and our previous research^77^ has demonstrated the feasibility of using the PolyA capture-based Visium technique on 3-week-old mouse IVD FF samples, providing a preliminary reference for ST studies in cartilage tissues.

In bone marrow, the abundance of different cell types varies by orders of magnitude,^168^ with non-hematopoietic cells, which include mesenchymal-origin stromal cells, accounting for less than 0.5% of the total cell count in adult mouse bone marrow.^169^ This makes the effective identification of rare cell types in the bone marrow particularly challenging. ST technology based on spatial barcodes typically lacks actual single-cell resolution, necessitating the use of deconvolution to determine the cell types and their proportions within each spot and to identify potentially present rare cell types.^170^ This step is often achieved by integrating scRNA-seq data or combining high-resolution H&E images, or through reference-free methods. Given that scRNA-seq is a powerful tool for identifying rare cell populations, and considering the abundance of bone marrow scRNA-seq datasets currently available,^15,84^ we emphasize the use of scRNA-seq to effectively identify rare cell types and further map them to the bone marrow niche, thus enhancing the analytical power of ST data. When identifying rare cell types in bone marrow using scRNA-seq, the critical roles of sample preparation, enrichment of rare cells, and the choice of library preparation platforms and marker genes must be fully considered.^171^ First, preparing scRNA-seq samples by enzymatic digestion of bone marrow tissues rather than by bone marrow aspiration or bone flushing enables the capture and identification of rare cells closely adhering to the bone surface, such as fibro-mesenchymal stromal cells, avoiding the detection bias of the mesenchymal stromal cell component towards adipocytes.^15,84^ Second, using prior knowledge of cell surface markers and anatomical locations to utilize flow cytometry or laser microscopy can significantly enhance the detectability of rare cells through cytometric or anatomic enrichment.^171^ For example, *Hoxb5*^*+*^ long-term hematopoietic stem cells constitute only about 0.001% of the nucleated cells in mouse bone marrow and are difficult to detect without enrichment.^172^ Third, using a plate-based full-length sequencing platform instead of a droplet-based short-read sequencing platform increases the scope and depth of RNA capture,^173^ improving the likelihood of detecting rare cell types and states in bone marrow. Fourth, methods such as CellSIUS^174^ and scPNMF^175^ have been developed to optimize the selection of marker genes, facilitating the identification of rare cell types in bone marrow. Additionally, integrating multiple bone marrow scRNA-seq datasets also assists in identifying rare stromal cell subpopulations.^176^ Furthermore, by integrating single-cell multi-omics data to enhance the comprehensive understanding of cellular heterogeneity, deep learning models such as MarsGT^177^ can more effectively infer and identify rare cells within the bone marrow.

Different deconvolution packages have their pros and cons. To improve the reliability and accuracy of data analysis, researchers have developed a joint predictive pipeline utilizing Seurat,^178^ CellTrek,^179^ and Cell2Location,^180^ successfully pinpointing the rare cell types, Cxcl12-abundant reticular cells in the bone marrow and *Pdgfra*^*+*^*Sca1*^*+*^ SSPCs in the periosteum.^12^ It is worth noting that deconvolution strategies based on scRNA-seq data depend on the quality and completeness of reference data and the compatibility between single-cell and spatial data. While we now have access to a wealth of bone marrow scRNA-seq datasets, we cannot guarantee that these datasets encompass all cell types and states. Packages that offer reference-free deconvolution capabilities such as CARD^181^ and STdeconvolve^182^ might help discover as-yet unidentified rare cell types. Conversely, for rare cell types with known marker genes, targeted ST technologies such as Xenium and GeoMx DSP can be used for detection.^183^ Additionally, studies with Seq-Scope^184^ and HDST^185^ have confirmed the capability of high-resolution spatial barcode-based ST technology to accurately identify and precisely locate rare cell types, suggesting the potential value of applying higher resolution technologies to bone marrow ST research.

### Prospects for the application of ST to the musculoskeletal system

ST has already contributed to substantial progress in the study of the musculoskeletal system, and we believe there is still significant potential for further advances in this field. Fracture is a common and frequent disorder of the musculoskeletal system, with approximately 5% to 10% of fracture patients experiencing delayed healing or non-union.^186^ The study by Rios et al.^120^ was the first to apply ST to human bone. However, existing ST studies on fractures, including this one, remain limited to analyses at only one or two specific time points.^119–121^ Comprehensive single-cell spatiotemporal transcriptomic maps covering the entire healing process of fractures at different anatomical sites are still lacking. Conducting combined scRNA-seq and ST analysis at different fracture sites and at different stages of the fracture-healing process could help us deepen our understanding of the mechanisms behind fracture healing and aid in the development of strategies to promote healing. Osteoporosis increases the risk of fractures and is associated with many skeletal diseases.^187^ The 3D gene expression atlas of trabecular and cortical bone under the influence of osteoporosis remain to be drawn. Lumbar disc herniation, often caused by intervertebral disc degeneration, is a common cause of lower back pain and chronic disability in the elderly, but its pathological mechanisms are not yet fully understood.^188^ Recently, Chen et al.^77^ constructed a spatial transcriptome atlas of the IVD without degeneration, while the spatial expression patterns of genes in the NP, AF, and CEP under intervertebral disc degeneration conditions still need further mapping. Additionally, athletes and fitness enthusiasts are often plagued by overuse injuries.^189^ In the future, using ST to reveal the response mechanisms underlying the spatial expression patterns of genes in tissues such as tendons and ligaments under different mechanical loads could help in formulating scientific training strategies and preventing injuries.

### The 3D landscape of the transcriptome

The spatial heterogeneity of bones, bone junctions, and skeletal muscles is not limited to two-dimensional planes but is also manifested in complex three-dimensional structures such as the network of trabecular bones, the varying cell density and collagen fiber arrangement from the superficial to deep layers of joint cartilage, and the intricate 3D patterns of blood vessels and nerve distributions within muscles. While current ST methods primarily focus on acquiring data in two-dimensional planes, accurately mapping and analyzing 3D structures are more complex and challenging. Tomo-seq processes RNA-seq for slices taken along the X, Y, and Z axes of three identical samples to reconstruct the 3D spatial transcriptome landscape by integrating data across these axes.^23^ Combining Tomo-seq and LCM, Geo-seq also has the capability of transcriptomic 3D reconstruction.^24^ Using innovative physical micro-sectioning techniques and algorithms similar to CT reconstruction, STRP-seq successfully reconstructs 3D gene expression patterns.^190^ STARmap performs high-resolution in situ imaging of target RNA in thick tissue slices, enabling 3D gene expression analysis of tissue samples.^54^ STARmap PLUS extends this capability to map transcripts and proteins in 3D.^191^ Electro-seq offers a potentially-powerful tool for characterizing cell states and developmental trajectories in electrogenic tissues such as skeletal muscles, incorporating chronic electrophysiological recordings while constructing 3D transcriptome landscapes.^55^ Open-ST can reconstruct virtual tissue blocks up to 350 μm thick, providing a 3D view of gene expression, cell types, and local signal transmission.^192^

Building 3D transcriptome landscapes from merely two-dimensional slice data involves overcoming challenges in data alignment and integration. Yet, the implementation of such strategies allows the construction of volumetric gene expression atlases to no longer be confined to specific ST methods. Zeira et al.^193^ introduced PASTE, which considers both transcriptional similarity and spatial proximity, analyzing consecutive 2D slice data to resolve the transcriptomic state of entire tissues in 3D space. Jones et al.^194^ proposed GPSA, which aligns spatial coordinates of sample data across tissue slices and data modalities using a common coordinate system, utilizing sparse 2D slices to construct a 3D atlas.

Accurate parsing and robust reconstruction of 3D structures will further enhance our understanding of internal interactions and functional dynamics within complex biological systems, especially the spatial relationships at the cellular and molecular levels and their implications for disease progression and tissue regeneration.

### Spatial multi-omics and spatiotemporal omics

Spatial multi-omics is a cutting-edge frontier that integrates various omics layers, including but not limited to epigenomics, transcriptomics, proteomics, and metabolomics, within a spatial context to provide multidimensional data, comprehensively uncovering the intricate micro-mechanisms of life processes.^195^ The development of spatial single-omics has propelled the continuous progress and innovation of spatial multi-omics. Typically, spatial multi-omics landscapes are constructed by using different spatial single-omics methods on adjacent slices. However, even adjacent slices can exhibit heterogeneity in spatial structure and cellular composition, and spatial resolutions may vary across different single-omics methods.^195^ DBiT-seq^68^ was the first technology to enable spatial multi-omics sequencing on the same slice,^196^ using a microfluidic deterministic barcoding strategy to detect the entire transcriptome and dozens of targeted proteins. Similarly, spatial-CITE-seq^197^ also maps both transcriptomes and proteins but targets up to hundreds of proteins. As previously mentioned, STARmap PLUS^191^ has the ability to perform 3D mapping of both transcripts and proteins. Combining CITE-seq with spatial barcode-based ST technologies, SM-Omics^198^ and SPOTS^199^ further realize spatial multi-omics, although with limited spatial resolution. Spatial ATAC–RNA-seq^200^ and Spatial CUT&Tag–RNA-seq^200^ analyze chromatin accessibility or histone modifications alongside the transcriptome, allowing for comprehensive studies on the complex processes of gene expression regulation. Dedicated frameworks like SpatialData^201^ have been developed for spatial multi-omics data. These frameworks greatly facilitate access to, sharing, and analysis of large-scale multidimensional data, providing powerful tools to overcome the limitations and challenges of traditional spatial omics data processing.

The journal “Nature” listed spatial multi-omics technologies as one of the top seven technologies to watch in 2022.^202^ Spatial multi-omics has begun to provide multi-omics landscapes with tissue context for projects like the Human Cell Atlas, aiming to build a detailed reference map of human cell types and states, significantly improving our understanding of the structure and function of human cells.^203^ However, spatial multi-omics is still in its developmental stage. For genomics and transcriptomics, the prevalent use of short-read sequencing technologies limits the detection of genetic variations and spatial alternative splicing.^204^ In proteomics, strategies that simultaneously offer high throughput and high spatial resolution still need further development. For metabolomics, constrained as it is by specific sample preparation methods, it is challenging to integrate with other spatial omics data on the same slice.^195^ Spatial epigenomics provides valuable data on gene regulation and personalized medicine, yet the simultaneous detection of DNA methylation, chromatin accessibility, and histone modifications on the same slice to finely and comprehensively delineate epigenetic features remains to be realized.^200^ Furthermore, the integration and analysis of multi-omics data present unprecedented challenges to existing bioinformatics tools. Lastly, high spatial resolution and true single-cell resolution, coupled with low cost and high throughput, remain the goals we strive for.

Currently, research on ST, especially applied to bone tissue in the musculoskeletal system, is indeed very limited. The decalcification process in bones typically leads to RNA degradation,^12^ which is one of the main limitations for conducting ST research in bone tissues; however, this process has little impact on the antigenicity of proteins in the sample, thus not hindering subsequent antibody-based spatial proteomics analyses.^205^ There exists a nonlinear complex relationship between mRNA and the proteins translated from it, often leading to discrepancies in their levels of expression.^206^ It is noteworthy that, compared to proteomic data, transcriptomic data tend to present a more complex landscape, whereas the proteome appears more stable and straightforward.^207^ Therefore, spatial proteomics analysis provides us with more direct and authentic insights into cellular functions and states.^208^ A recent study^15^ utilized scRNA-seq and co-detection by indexing, a spatial proteomics technique based on multiple antibodies, to analyze the cellular composition and spatial landscape of the human bone marrow niche, focusing on the enrichment and analysis of low-abundance non-hematopoietic cells. By integrating single-cell transcriptomic data with spatial proteomic data, intercellular signaling and spatial proximity within the bone marrow were revealed. Additionally, the co-detection by indexing maps helped uncover the expansion of mesenchymal stromal cells and their spatial proximity and co-enrichment with leukemic blasts in acute myeloid leukemia.^15^ To our knowledge, this study is the first to construct a spatial multi-omics map of the human bone marrow through the integration of multi-omics data across different dimensions, providing a key multi-omics perspective for comprehensively analyzing the human bone marrow niche. Based on this, we emphasize the significant potential and value of constructing spatial multi-omics landscapes through the combination of spatial single-omics and other omics without spatial resolution, rather than relying on the premise that all omics data have spatial resolution. Although this article mainly focuses on ST, it is undeniable that spatial proteomics may offer a more practical option for spatial multi-omics research in bone tissues.

Spatiotemporal omics not only focuses on the distribution and function of cells and molecules in space but also considers how these characteristics change over time. Currently, ST can only capture the transient states of continuous processes, limiting our deep understanding of the dynamic evolution of physiological and pathological processes. Methods to construct a spatiotemporal transcriptome atlas rely on sampling multiple specimens at different time-points within the same physiological or pathological process. Continuous molecular analysis of live cells to decipher cell dynamics has been a focus area for researchers. Live-seq,^209^ for the first time, enabled continuous observation and analysis of single-cell transcriptomic spectra and downstream molecules in the same cell without causing significant cell perturbations. Live-seq provided temporal resolution to scRNA-seq. Future enhancements to Live-seq that add tissue spatial context, such as combining it with ROI-selection-based ST technologies, will greatly advance the development of spatiotemporal transcriptomics. Additionally, continuous advances in mass spectrometry will significantly enhance our understanding and exploration of the proteome, peptidome, and metabolome in spatiotemporal multi-omics studies.^210^

### The application of AI in ST

The multidimensional data from ST, which include complex gene expression matrices, 2D and even 3D spatial information, and histological images, demand high capabilities in data processing and analysis.^211^ As ST technologies continue to evolve, moving toward higher-dimensional spatial multi-omics and spatiotemporal omics, the volume and complexity of data are set to increase. This progression poses new challenges for data handling and analysis, but it also creates significant opportunities for AI, particularly for deep learning (DL) strategies within machine learning. Currently, DL has been extensively applied to various aspects of ST analysis. For instance, models like SpaGCN^212^ and stLearn^213^ can effectively cluster data based on gene transcription features and spatial distributions. SpaGCN^212^ and STAGATE^214^ can efficiently identify spatially-variable genes. Tools such as stPlus^215^ and Tangram^216^ integrate single-cell and spatial transcriptomics data to impute missing genes. stLearn^213^ and GCNG^217^ leverage DL methods to identify critical ligand–receptor pairs, thereby inferring intercellular communication networks. For sequencing-based ST technologies, methods like DSTG^218^ and Tangram^216^ can predict the proportions of different cell types at mixed cell loci, achieving deconvolution of cell types. For imaging-based ST technologies, tools such as Baysor^219^ and JSTA^220^ effectively segment cells. Deep learning models such as SAUCIE,^221^ DESC,^222^ and CarDEC^223^ have been developed to overcome batch effects in scRNA-seq. Although methods like SEDR^224^ and STAGATE^214^ have limitations in handling batch effects in ST, particularly in not considering histological images, they represent early attempts and provide preliminary solutions to alleviate batch effects in this field. We believe that the continuous innovations in AI, especially DL, coupled with the further opening and sharing of ST data, is crucial for fully unlocking the potential of these data. This will provide unprecedented insights into the complex molecular mechanisms of health and disease.

## Conclusion

This article outlines the development of ST data acquisition technologies, highlighting new technologies, advances, and representative methods, providing a concise workflow for incorporating ST into musculoskeletal system research, summarizing the applications of ST in the musculoskeletal system, and discussing the challenges and prospects for ST itself and its application in the musculoskeletal system. When conducting ST studies, the sample preparation and permeation processes of the musculoskeletal system, especially bone, cartilage, and tendon tissues, as well as the effective identification of rare cell types in the bone marrow, remain challenging. As capabilities in sample processing, tissue permeabilization, and rare cell identification continue to advance, along with the deep application of AI in ST, the field of ST is constantly evolving towards spatial multi-omics and spatiotemporal omics, continuously advancing and innovating. This progress is also reflected in the transition from 2D transcriptomic data to 3D transcriptomic landscapes, providing us with a more comprehensive and in-depth new perspective on understanding gene expression patterns within organisms. In the future, further application of ST to problems such as fractures and lumbar disc herniation will push research on the musculoskeletal system into a new stage. We believe that the data acquisition and analysis technologies of ST will continue to develop rapidly, bringing continuous breakthroughs to research into the musculoskeletal system.
